# Differential Expression of One‐Carbon Pathway Enzyme ALDH1L1 Is Linked to Tumorigenicity of Low‐Grade Bladder Cancer Cells Through Metabolic Reprogramming

**DOI:** 10.1002/cam4.71291

**Published:** 2025-10-10

**Authors:** Halle M. Meyers, Jaspreet Sharma, Amira A. Abdellatef, Mikyoung You, David Raines, Kyle C. Strickland, Susan Sumner, Blake R. Rushing, Natalia I. Krupenko, Sergey A. Krupenko

**Affiliations:** ^1^ Nutrition Research Institute UNC‐Chapel Hill Kannapolis North Carolina USA; ^2^ Department of Nutrition UNC‐Chapel Hill Chapel Hill NC USA; ^3^ Department of Food and Nutrition and the Convergence Center for Green Anti‐Aging Research Mokpo National University Republic of Korea; ^4^ Labcorp Durham NC USA; ^5^ Duke University Medical Center, Duke Cancer Institute, Department of Pathology Durham NC USA

**Keywords:** ALDH1L1, one‐carbon metabolism, RT4 bladder cancer cells, untargeted metabolomics, xenograft tumors

## Abstract

**Background:**

RT4 bladder cancer cell line, derived from a nonmuscle‐invasive low‐grade subtype, is one of the few neoplastic cell lineages that maintain high expression of the candidate tumor suppressor ALDH1L1. Here, we investigated how differential ALDH1L1 expression affects cellular characteristics and tumorigenicity of RT4 cells as well as tumor metabotypes.

**Methods:**

We characterized RT4 cells and two shRNA clones (sh506/low ALDH1L1 expression; sh572/ALDH1L1 is lost) for proliferation, migration, clonogenic capacity, and mitochondrial respiration. We have further evaluated the tumorigenic potential of RT4 cells and the two clones in nude mice and compared metabotypes of derived tumors using untargeted metabolomics.

**Results:**

Both clones with diminished ALDH1L1 expression exhibited increased proliferation rates with doubling times of 19.4 h (sh506) and 23.2 h (sh572) versus 36.3 h for RT4 cells. Downregulation of ALDH1L1 expression also enhanced motility and clonogenic capacity. Proliferation and clonogenic capacity were highest for the sh506 clone (low ALDH1L1 expression), while motility was strongest for the sh572 clone (complete ALDH1L1 loss). Both clones showed altered energy metabolism, as indicated by a reduced basal oxygen consumption rate and enhanced maximal respiration rate following oligomycin treatment. Mouse xenograft tumors derived from ALDH1L1‐deficient RT4 clones were significantly larger than RT4 cell‐derived tumors. Of note, complete ALDH1L1 loss (sh572 clone) was less advantageous for tumor growth than the partial loss of the protein (sh506 clone). Untargeted metabolomics has shown that tumors with downregulated ALDH1L1 have altered the metabolism of fatty acids, amino acids, CoA, and acylcarnitines. Alterations in several key pathways, including glutathione metabolism (sh506), and TCA cycle (sh572), depend on the extent of ALDH1L1 downregulation.

**Conclusions:**

Our study underscores ALDH1L1 as a key metabolic regulator of proliferation, migration, and tumorigenicity in RT4 bladder cancer cells, suggesting that retaining low ALDH1L1 expression can provide a metabolic advantage for growth of aggressive tumors.

## 1 Introduction

Bladder cancer, a common malignancy that typically affects older adults, is a global health concern leading to more than 220,000 deaths each year worldwide [1]. Major risk factors for bladder cancer include tobacco use, exposure to occupational carcinogens, chronic inflammation of the bladder, and genetic susceptibility [2]. Urothelial carcinoma is the most prevalent histological subtype of bladder cancer [1, 3]. It is characterized by abnormal cell growth in the bladder's urothelial lining and exhibits a broad spectrum of clinical and pathological features, ranging from nonmuscle‐invasive tumors to aggressive muscle‐invasive types with a strong tendency to metastasize [4]. Although low‐grade urothelial carcinoma has a lower risk of progressing to invasive disease, its high recurrence rate and requirement for continuous monitoring pose a significant clinical challenge and contribute considerably to a healthcare burden [5].

At the molecular level, bladder cancer is marked by genetic alterations and dysregulation in key signaling pathways that drive uncontrolled cell proliferation, survival, and invasion [6]. These include mutations in the fibroblast growth factor receptor‐3 (FGFR‐3) [7], chromosome 9 deletions [8], oncogenic HRAS [9], and dysregulation of the phosphatidylinositol 3‐kinase (PI3K)/AKT signaling [10, 11]. Further, metabolic reprogramming, which provides the energy and biosynthetic precursors necessary for cancer progression, is increasingly recognized as a hallmark of bladder cancer [12]. Dysregulation in key metabolic pathways such as glycolysis and the TCA cycle, as well as altered lipid metabolism, are prominent features in bladder cancer cells [13]. Such metabolic reprogramming facilitates malignant processes in response to the exposure to environmental carcinogens by promoting the rapid proliferation and invasiveness of tumor cells. The importance of specific metabolic pathways in bladder cancer tumorigenesis and progression underscores the need for a deeper understanding of the role of key metabolic enzymes that regulate these pathways [12]. This knowledge is critical for bladder cancer prevention and the identification of suitable therapeutic targets for treating this cancer.

One‐carbon metabolism, also known as folate metabolism, is involved in several key pathways within the cell, including nucleotide biosynthesis, methyl group biogenesis, and maintenance of redox balance [14, 15]. Active folate metabolism is also required for cancer cells, with several enzymes of these pathways being commonly upregulated in malignancies [15]. Recent studies have suggested that one of the folate enzymes, aldehyde dehydrogenase 1 family member L1 (ALDH1L1), has an important function in tumor biology, as it affects cellular metabolism, proliferation, and migration processes essential for cancer progression [16, 17, 18, 19]. ALDH1L1 functions in a catabolic fashion, oxidizing the folate‐bound formyl group to carbon dioxide [20]. The ALDH1L1 reaction (10‐formyltetrahydrofolate + NADP^+^ + H_2_O → tetrahydrofolate + NADPH + H^+^) facilitates the elimination of one‐carbon groups from the folate cycle, thereby limiting their availability for folate‐dependent biosynthetic pathways, including purine and thymidylate synthesis [21]. Through this regulatory role, ALDH1L1 acts as a metabolic checkpoint, modulating anabolic processes essential for cell proliferation. ALDH1L1 also contributes to the generation of NADPH, which is involved in maintaining cellular redox balance (reviewed in [21]). Since NADPH is essential for antioxidant defense systems such as glutathione recycling, as well as for fatty acid and cholesterol biosynthesis (reviewed in [22]), ALDH1L1 likely has a broader role in cellular homeostasis beyond its direct function in folate pathways. Although localized in the cytosol, ALDH1L1 might also influence mitochondrial metabolism by modulating the availability of cytosolic one‐carbon units [19], which can impact mitochondrial formate production, ATP generation, and the redox balance between the cytosol and mitochondria. Altered ALDH1L1 expression may disrupt this cross‐compartmental metabolic coordination, potentially impairing mitochondrial function and energy metabolism.

ALDH1L1 expression varies between tissues [16], but the enzyme is frequently downregulated in malignant tumors and commonly silenced in cancer cell lines (reviewed in [21]). Overall, reduced expression of ALDH1L1 has been linked to tumor progression [21]. Interestingly, though, a bladder cancer cell line, RT4, established from a transitional cell papilloma of a 63‐year‐old Caucasian male, is a notable exception to this trend. RT4 cells represent low‐grade, nonmuscle‐invasive bladder cancer, which is characterized by frequent recurrence, but a relatively low risk of progression compared to muscle‐invasive bladder cancer [23, 24, 25]. RT4 cells express high levels of ALDH1L1 [26], which raises the question of how certain neoplastic cell types evade ALDH1L1‐dependent proliferation control. There is also a question of whether the loss of ALDH1L1 expression in RT4 cells is associated with aggressiveness, invasion, and/or poor differentiation. This would align with ALDH1L1's role in normal cellular metabolism and the frequent association of the loss of enzyme expression with more aggressive cancer phenotypes [21]. Overall, RT4 cells provide an opportunity to investigate the effect of ALDH1L1 knockout on metabolism, proliferation, and tumorigenicity.

We previously reported that downregulation of ALDH1L1 expression in RT4 cells is associated with strong effects on cellular metabotype [26]. Here, we investigated how ALDH1L1 loss and associated metabolic alterations affect the proliferation of RT4 cells and their tumorigenicity in a xenograft mouse model. In this study, we compared original RT4 cells exhibiting high ALDH1L1 expression with two stable shRNA clones which express low and undetectable levels of ALDH1L1. This approach enabled us to gain further insight into the role of ALDH1L1 in regulating tumor aggressiveness and assess the underlying metabolic dysregulations.

## 2 Materials and Methods

### 2.1 Cell Culture

The human bladder cancer cell line, RT4, was purchased from American Type Culture Collection (ATCC, Manassas, VA, USA) and cultured for no more than 5 sequential passages. Cells were grown in McCoy's 5A medium (Thermo Fisher Scientific, Waltham, MA, USA) supplemented with 10% fetal bovine serum (FBS, Bio‐Techne, Minneapolis, MN, USA) and 1% antibiotic‐antimycotic (Thermo Fisher Scientific) at 37°C in a humidified atmosphere with 5% CO_2_. ALDH1L1‐deficient RT4 clones were generated by shRNA as we previously described [26]. The original RT4 cell line and derived clones were mycoplasma‐free and retained the same morphology throughout the study.

### 2.2 Doubling Time

RT4 cells were seeded at a uniform density of 20,000 cells per plate and harvested 48 h later. Cells were stained with Trypan Blue and counted using a Countess II FL automated cell counter (Thermo Fisher Scientific, formerly Life Technologies). Doubling time was determined from the growth curves using the data generated from three independent experiments, each experiment with three technical replicates.

### 2.3 Cell Proliferation Assay

Cell viability was assessed using Promega MTT cell proliferation assay. Cells were seeded in 96‐well plates at a density of 5 × 10^3^ cells per well. MTT was added at specific time points, and plates were further processed according to the manufacturer's instructions. Absorbance was measured at 570 nm using a Victor X5 Multimode Plate Reader (PerkinElmer Life Sciences, Waltham, MA).

### 2.4 EdU Staining Assay

EdU staining was used to evaluate the proliferation of RT4 clones, according to the Click‐iT Plus EdU Alexa Fluor 555 Imaging Kit (Invitrogen, Thermo Fisher Scientific). RT4 clones were incubated with EdU at a concentration of 10 μM for 4 h. Cells were then washed with PBS and fixed in 3.7% paraformaldehyde for 20 min. After fixation, cells were rinsed with PBS and permeabilized using 0.5% Triton X‐100 for an additional 20 min. Subsequently, cells were washed three times with 5% BSA in PBS (Blocking buffer), and then the Click‐iT reaction mixture was applied to the samples for 30 min at room temperature, away from light. Finally, the cells were washed and stained with DAPI solution (Invitrogen). Fluorescence images were captured and analyzed using a Carl Zeiss LSM 700 confocal microscope. At least five representative images were taken for each group. EDU‐positive cells were analyzed using ImageJ software, version 1.54 g, and GraphPad Prism software, version 10.4.0 (GraphPad Software, San Diego, CA). This experiment was independently repeated three times to ensure reproducibility.

### 2.5 Real‐Time Quantitative Cell Analysis (RTCA)

Experiments were performed at 37°C in a humidified atmosphere with 5% CO_2_ using an xCELLigence RTCA DP system (ACEA Biosciences) following the manufacturer's instructions. Cell proliferation was continuously monitored in real time using an E‐plate 16 (ACEA Biosciences), as we previously described [27]. Background impedance readings for each well were recorded using cell‐free medium (100 μL per well) after preincubation at room temperature for 30 min. Cells were seeded in each well in 100 μL cell suspension across a concentration range of 5 × 10^3^ to 8 × 10^4^ cells/well and allowed to attach for 30 min at room temperature. Plates were set into the system, and impedance readings from each well were automatically recorded every 15 min for the duration of the experiment.

### 2.6 Wound Healing Assay

Cells (2 × 10^5^ cells/well) were seeded in a 12‐well plate and cultured for 24 h to form a monolayer (80%–90% confluency). Scratches were made using a sterile pipette tip, followed by washing with PBS to remove detached cells and debris. Fresh medium (1 mL) was then added to each well. Images were captured at 0 h and 48 h to assess the extent of wound healing. Wound closure was calculated using the following equation: wound closure (%) = [1 − (cell‐free area at 48 h/cell‐free area at 0 h)] × 100, with the cell‐free area measured immediately after the scratch (0 h) and 48 h later. The wounded area was quantified manually using ImageJ software, version 1.54 g.

### 2.7 Colony Formation Assay

Cells were counted (2 × 10^3^ cells/well), seeded in triplicate in 6‐well plates, and incubated for 2 weeks in a standard medium at 37°C with 5% CO_2_. Colonies were fixed with an ice‐cold methanol‐acetone mixture (1:1) and stained with 0.5% crystal violet (Sigma). Images were captured and analyzed using a Keyence BZ‐X700 fluorescence microscope and software.

### 2.8 Mitochondrial Stress Test

The cellular oxygen consumption rate (OCR) was assessed using an XV96 Seahorse Metabolic Flux Analyzer (Agilent Seahorse Technologies, Santa Clara, CA, USA). In brief, RT4 cells were seeded in XF96 Seahorse cell culture plates at a density of 4 × 10^4^ cells per well and incubated overnight. Cells were pre‐incubated in assay media (serum‐free RPMI‐1640 media with 10 mM glucose, 2 mM glutamine, and 1 mM pyruvate, without bicarbonate, pH 7.4) in a nonCO_2_ incubator for 1 h before analysis. OCRs were measured after the sequential addition of oligomycin (O; 1.0 μM), carbonyl cyanide‐4‐(trifluoromethoxy) phenylhydrazone (FCCP; 1.0 μM), and rotenone/antimycin A (R; 0.5 μM). Data were normalized by the total protein measured using a bicinchoninic acid protein assay (Thermo Fisher Scientific) and expressed as relative OCR.

### 2.9 Subcutaneous Xenograft Tumor Model

All animal experiments were conducted in compliance with the National Institutes of Health's “Guide for Care and Use of Laboratory Animals” and were approved by the Institutional Animal Care and Use Committee guidelines (IACUC) at the North Carolina Research Campus (NCRC), under protocol No. 21–010. Mice were housed under a standard 12:12‐h light–dark cycle and provided with ad libitum access to standard chow. To generate subcutaneous (sc) RT4 tumors, parental RT4, sh506, and sh572 (2 × 10^6^ cells) were suspended in 100 μL of PBS containing 50% Matrigel (Corning) and injected bilaterally into the flanks of Athymic Nude Mice (Envigo; 5–6‐week‐old males). A total of 18 mice were used: RT4 (*n* = 6), sh506 (*n* = 6), and sh572 (*n* = 6). Tumors were measured weekly with digital calipers; tumor volume was calculated using the formula: tumor volume = (L × W^2^)/2 (“L” is length and “W” is width). No attrition was observed throughout the experiment. After 12 weeks, the mice were sacrificed, and tumors were excised and flash‐frozen at −80°C, with portions of the tumors preserved in 10% neutral‐buffered formalin for further analysis.

### 2.10 Ki‐67 Immunohistochemistry and Analysis

Fixed tissues were embedded in paraffin, and tissue blocks were sectioned at 5 μm and mounted on glass slides. Slides were stained with hematoxylin and eosin (H&E) per standard protocols. Slides were deparaffinized, rehydrated, and treated with 3% hydrogen peroxide. Antigen retrieval was performed by boiling the sections for 10 min in 0.1 M citrate buffer antigen retrieval solution (pH 6.0). Nonspecific antibody binding was blocked using 2% non‐fat milk in TBST for 30 min. Tumor sections were stained with Ki‐67 antibody (Cell Signaling Technology, #9027S). H&E‐stained sections and immunostained slides were examined by a board‐certified anatomic pathologist (KCS). Digital photomicrographs were obtained in triplicate for each slide using an Olympus BX46 microscope at high magnification (40×), and the number of positive and negative nuclei was manually counted from each image to determine the percent Ki‐67 proliferation index.

### 2.11 Metabolite Extraction

Metabolites were extracted from tumors using the previously described protocol [28]. Tumor samples (27 total, 100–200 mg of flash‐frozen tissue each) included: RT4‐derived tumors, *n* = 5; sh506‐derived tumors, *n* = 10; and sh572‐derived tumors, *n* = 12. Tumor weight and volume were measured prior to metabolomics processing, and selected samples were those that were closest to the group mean values. Additionally, we performed western blot analysis on all selected samples to confirm consistent ALDH1L1 expression within each group and homogenized them using ceramic beads in a solution of 80% methanol and 20% water at a volume of 5 μL/mg of tumor tissue. Homogenates were clarified by centrifuging at 16,000 *g* for 10 min at 4°C, and 100 μL of the supernatant was dried using a speedvac. Quality control study pools (QCSPs) were generated by combining an additional 20 μL of the supernatant from each sample, aliquoting at 100 μL, and then dried using a speedvac. Samples were reconstituted by adding 100 μL of a solution containing 95% water and 5% methanol, while vortexing at 5000 rpm for 10 min. Final reconstituted extracts were centrifuged at 16,000 *g* for 10 min at 4°C, and 5 μL was injected for metabolomics analysis by ultra‐high‐performance liquid chromatography‐high resolution mass spectrometry (UHPLC‐HRMS).

### 2.12 UHPLC‐HRMS Analysis

Untargeted metabolomics analysis was performed using a Vanquish UHPLC connected to a Q Exactive HF‐X Hybrid Quadrupole‐Orbitrap Mass Spectrometer (Thermo Fisher Scientific). Instrument method parameters were set up using previously described protocols [26, 29, 30, 31]. Samples were loaded in an autosampler in random order with QCSP and blank injections interspersed after every six study samples. Separation of metabolites was performed using a HSS T3 C18 column (2.1 × 100 mm, 1.7 μm, Waters Corporation) at 50°C. Mobile phases consisted of (A) water +0.1% formic acid (v/v) and (B) methanol +0.1% formic acid (v/v). Chromatographic separation was performed using a gradient that started at 1% B and increased to 100% B in 16 min, then held for 4 min, with a constant flow rate of 400 μL/min throughout. The full scan range was set to 70–1050 *m*/*z* and MS/MS data was acquired using a data‐dependent acquisition mode that fragmented the top 20 most abundant ions. Preprocessing of raw instrument files was performed using Progenesis QI (version 2.1, Waters Corporation, Milford, MA, USA). Peaks with a higher average abundance in the blanks compared to the QCSPs were filtered out to remove background. Normalization was performed in Progenesis QI using the “normalize to all” feature [32]. Multivariate analyses, including principal component analysis (PCA) and orthogonal partial least squares–discriminant analysis (OPLS‐DA), were performed using SIMCA 16 (Umetrics, Umeå, Sweden). PCA visualization of the processed data demonstrated that the QCSPs were clustered and in the center of the study samples [33].

### 2.13 Metabolite Identification/Annotation

Peaks were matched to metabolites using an in‐house reference standard library and public databases (NIST, METLIN, HMDB). Peaks were matched to metabolites based on exact mass (MS, < 5 ppm), MS/MS fragmentation pattern (similarity score > 30%), isotope pattern (similarity score > 90%), or retention time (RT, only for in‐house library standards, ±0.5 min). Communication of the evidence for each match was performed using a previously described ontology system [26, 29, 31]. Labels include: OL1 (match to in‐house library by MS, MS/MS, and RT), OL2a (match to in‐house library by MS and RT), OL2b (in‐house match by MS and MS/MS), PDa (public database match by MS and MS/MS), PDc (public database match by MS and isotope pattern), and PDd (public database match by MS only). Compound names are based on purchased standards or names listed in public databases. Certain isomeric forms may not be distinguishable using this method. OL1, OL2a, OL2b, and PDa are equivalent to Metabolomics Standards Initiative (MSI) level 1 matches, whereas PDb, PDc, and PDd are equivalent to MSI level 2 matches [34].

### 2.14 Statistical Analysis and Pathway Enrichment

Statistical analyses for the in vitro and in vivo experiments were performed using an unpaired Student's *t* test (after confirming normal distribution) to assess statistical significance between two groups, or one‐way analysis of variance (ANOVA) for multiple‐group comparisons, followed by Tukey's post hoc test. A *p*‐value of less than 0.05 was considered statistically significant, with *p < 0.05; **p < 0.01, ***p < 0.001, and ****p < 0.0001. Data were analyzed using GraphPad Prism version 10.4.0. software (GraphPad Software, San Diego, CA).

For untargeted metabolomics, data were analyzed using Metaboanalyst 6.0 [35] or SIMCA 18.0 (Sartorius Stedim Data Analytics, AB, Umeå, Sweden). Principal Component Analysis (PCA) and Orthogonal Partial Least Squares–Discriminant Analysis (OPLS‐DA) multivariate statistics were conducted to assess group differences. Median peak abundance values were used to calculate fold changes. Student's *t* test was used for *p*‐value calculations.

The Enrichment Analysis module in MetaboAnalyst 6.0 was used for pathway analysis [35]. Input lists for the analysis included OL1 and OL2a metabolites with the *p* < 0.05 cutoff for the groups’ comparison.

## 3 Results

### 3.1 mRNA Expression of ALDH1L1 and Other Folate‐Related Enzymes in Cancer Cell Lines

**FIGURE 1:**
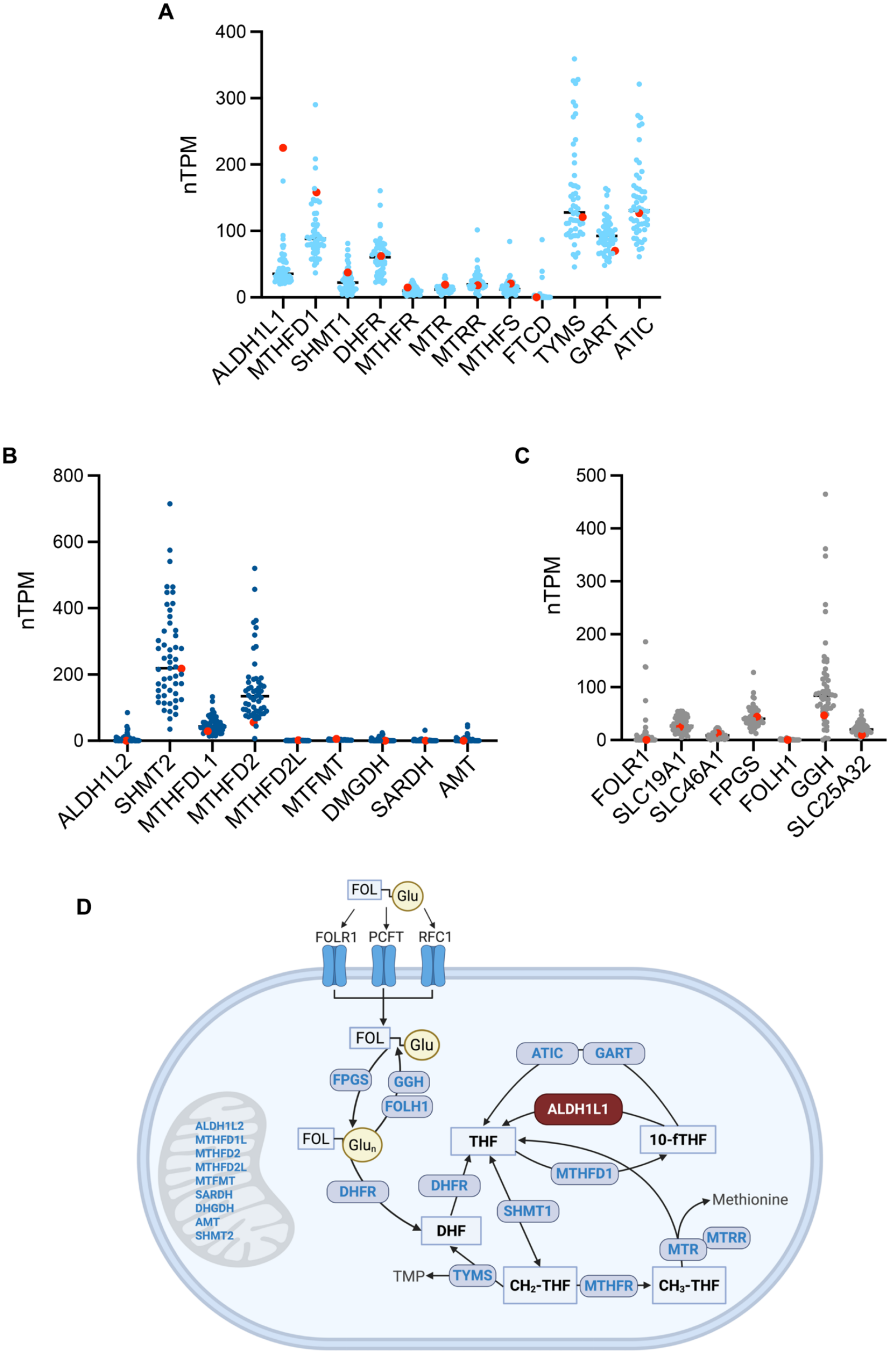
Expression of folate enzymes in a panel of cell lines with measurable ALDH1L1 mRNA. Levels of mRNA for cytosolic folate enzymes (A), mitochondrial folate enzymes (B) and folate transporters/enzymes involved in folate polyglutamylation (C) (from The Human Protein Atlas; each dot represents the mRNA levels for a single cell line; cell lines are listed in Table S1). *Red dots* indicate levels of mRNA for corresponding proteins in RT4 cells. (D) Disposition of enzymes in folate metabolism (focused on cytosolic folate pathways). Three cytosolic enzymes (FPGS, FOLH1, and GGH) are involved in folate polyglutamylation and the hydrolysis of polyglutamate tail. Three folate transporters are also included in the schematic. Mitochondrial enzymes are listed without the assignment to specific reactions. FOL‐Glu, folic acid; FOL‐Glu_n_, folate polyglutamate; DHF, dihydrofolate; THF, tetrahydrofolate; 10‐fTHF, and 10‐formyltetrahydrofolate. Abbreviations for protein names are based on the gene nomenclature as depicted in the Human Protein Atlas.

Analysis of the Human Protein Atlas database (https://www.proteinatlas.org/) shows that RT4 cells exhibit remarkably elevated levels of ALDH1L1 mRNA compared to other cancer cell lines (Figure 1A). To select cell lines for this analysis, a cut‐off threshold of 20 normalized transcripts per million (nTPM) was applied to define the lowest expression level of ALDH1L1 (the full list of 53 analyzed cell lines is shown in Table S1). We further evaluated the expression of other enzymes involved in folate metabolism in the same panel (Figure 1A–D shows the disposition of these enzymes in metabolic pathways). This analysis indicates that ALDH1L1 is one of the most highly expressed folate enzymes in RT4 cells.

### 3.2 Proliferation and Motility of ALDH1L1‐Deficient RT4 Cells

**FIGURE 2:**
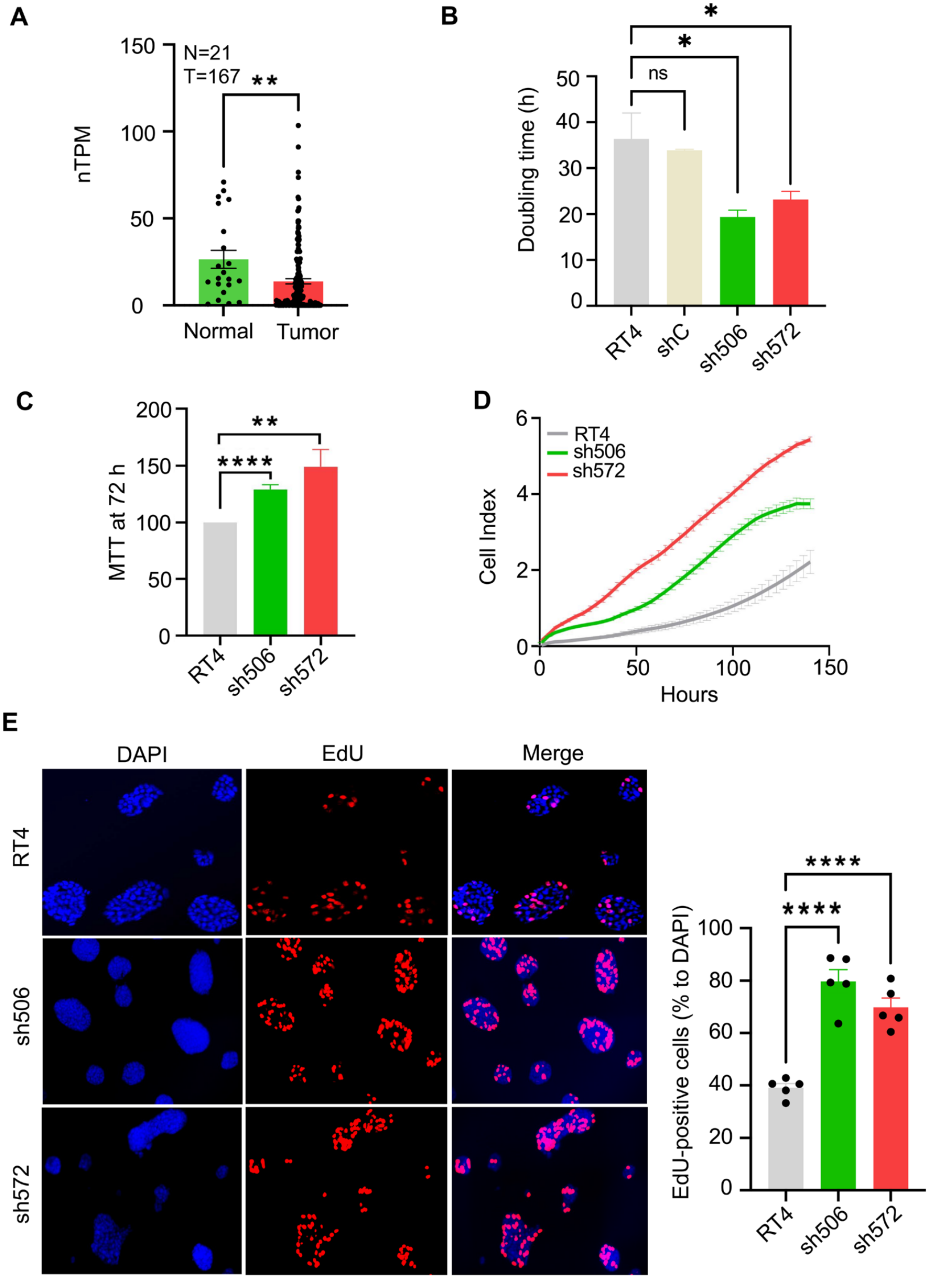
Effect of ALDH1L1 loss on cellular proliferation in ALDH1L1‐deficient RT4 clones. (A) Analysis of ALDH1L1 mRNA in bladder tumors and normal bladder tissues. (B) Doubling time of different RT4 clones. (C) MTT assay measuring proliferation at 72 h, comparing the different RT4 clones. (D) Real‐time proliferation index analysis of ALDH1L1‐deficient clones versus parental cells, conducted using an xCELLigence system (triplicate experiments with automated averaging of data points). (E) Left panel, representative images of the EdU staining assay in ALDH1L1‐deficient RT4 clones and parental cells after 4 h of EdU exposure. Right panel, quantification of the ratio of EdU + cells. Data represented as mean ± SEM of at least three independent experiments. ****, *p* < 0.0001; **, *p* < 0.01; *, *p* < 0.05 determined by unpaired Student's t‐test after confirming normal distribution (A), or by one‐way ANOVA for comparisons involving more than two groups, followed by Tukey's multiple comparison tests (B–E). Nonsignificant results were denoted as “ns”.

**FIGURE 3:**
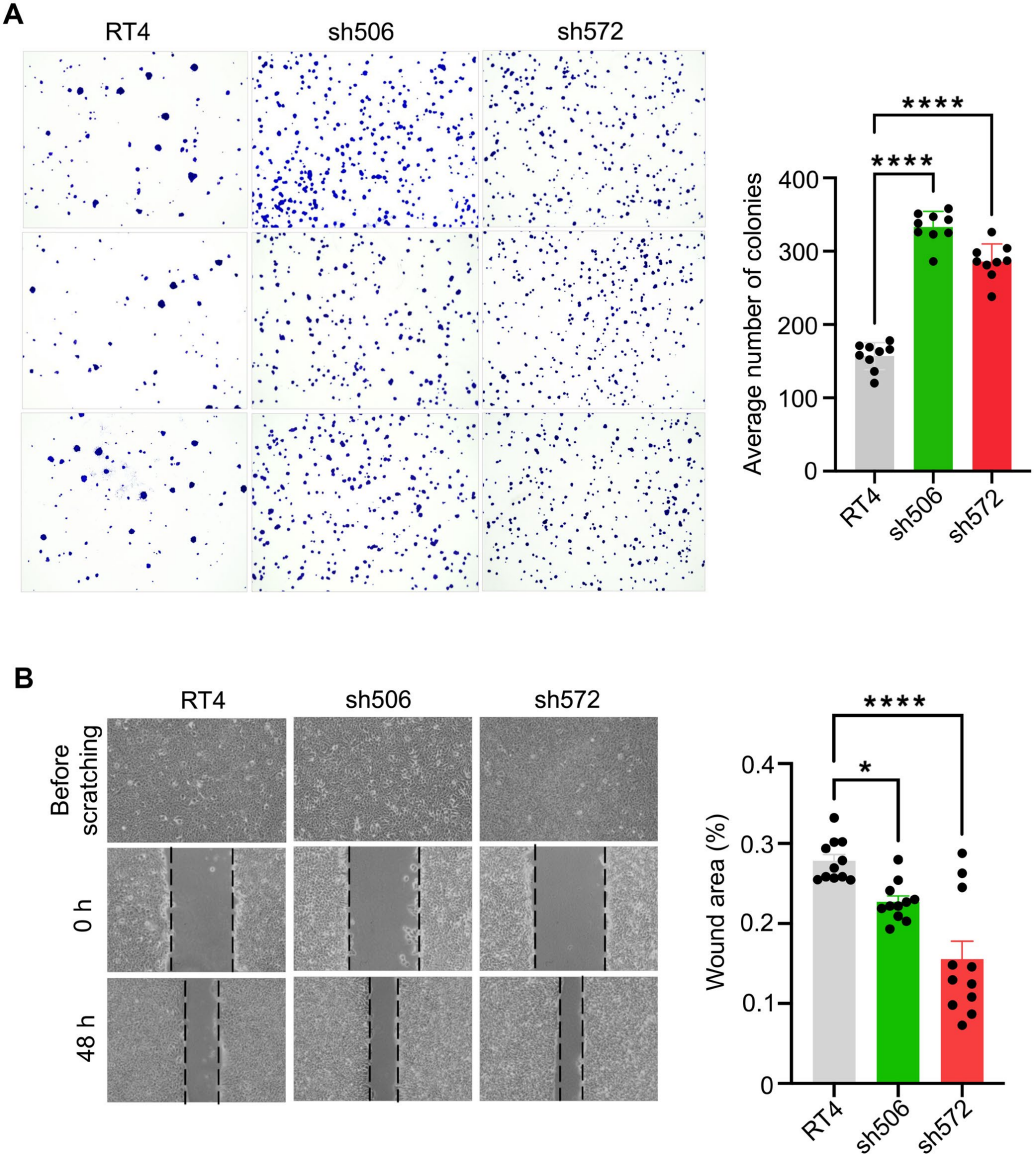
Effect of ALDH1L1 loss on the colony formation and migration abilities in ALDH1L1‐deficient RT4 clones. (A) Left panel, representative images of colony formation by ALDH1L1‐deficient RT4 clones after 14 days. Right panel, quantification of colony numbers, presented as the mean ± SEM of at least three independent experiments (*n* = 3). (B) Left panel, wound healing assay images showing migration of ALDH1L1‐deficient RT4 clones over a 48 h period. Right panel, bar graph representing the mean ± SEM of wound closure at 48 h measured in triplicate. Data show mean ± SEM; ****, *p* < 0.0001; *, *p* < 0.05, determined by one‐way ANOVA for comparisons involving more than two groups, followed by Tukey's multiple comparison tests (A, B).

Although average levels of ALDH1L1 mRNA are lower in bladder cancers than in normal bladder tissues, there is strong heterogeneity in the enzyme expression between patients (Figure 2A). This analysis was based on data sourced from the Human Protein Atlas database (https://www.proteinatlas.org/). Two outlier samples were excluded from the tumor group analysis because they fell outside the standard deviation range from the group mean. To assess the impact of ALDH1L1 downregulation on cellular proliferation and motility of RT4 cells, we utilized previously generated stable clones with reduced (sh506) and undetectable (sh572) ALDH1L1 expression, and control shRNA clone (shC) [26]. All clones were cultured in high‐folate medium (McCoy's) and low‐folate medium (RPMI), confirming reduced ALDH1L1 expression in the sh506 clone and complete knockout in the sh572 clone. Parental RT4 cells and the shC control clone showed comparable ALDH1L1 expression levels (Figure S1A–C). Both clones demonstrated significantly shorter doubling times than the parental RT4 cells (36.3 h vs. 19.4 h for sh506, *p* = 0.018, and 23.4 h for sh572, *p* = 0.044). No difference in doubling time was observed in these experiments between parental RT4 cells and shC clones(Figure 2B). MTT assay confirmed a higher proliferation rate in ALDH1L1‐deficient clones than parental RT4 cells (Figure 2C), and no noticeable difference in proliferation was observed between parental RT4 cells and shC clones, confirming the reliability of both controls (Figure S2). Real‐time monitoring of the proliferation index over 150 h further validated the increased proliferative capacity of these clones (Figure 2D); the finding was also supported by EdU staining and clonogenic assays (Figures 2E and 3A). Additionally, ALDH1L1 downregulation significantly enhanced the motility of sh506 and sh572 clones, as demonstrated by the wound healing scratch assay (Figure 3B). These data indicate that ALDH1L1 downregulation or silencing promotes cellular properties associated with increased proliferation and motility.

### 3.3 Metabolic Flux Analysis of ALDH1L1‐Deficient RT4 Cells

**FIGURE 4:**
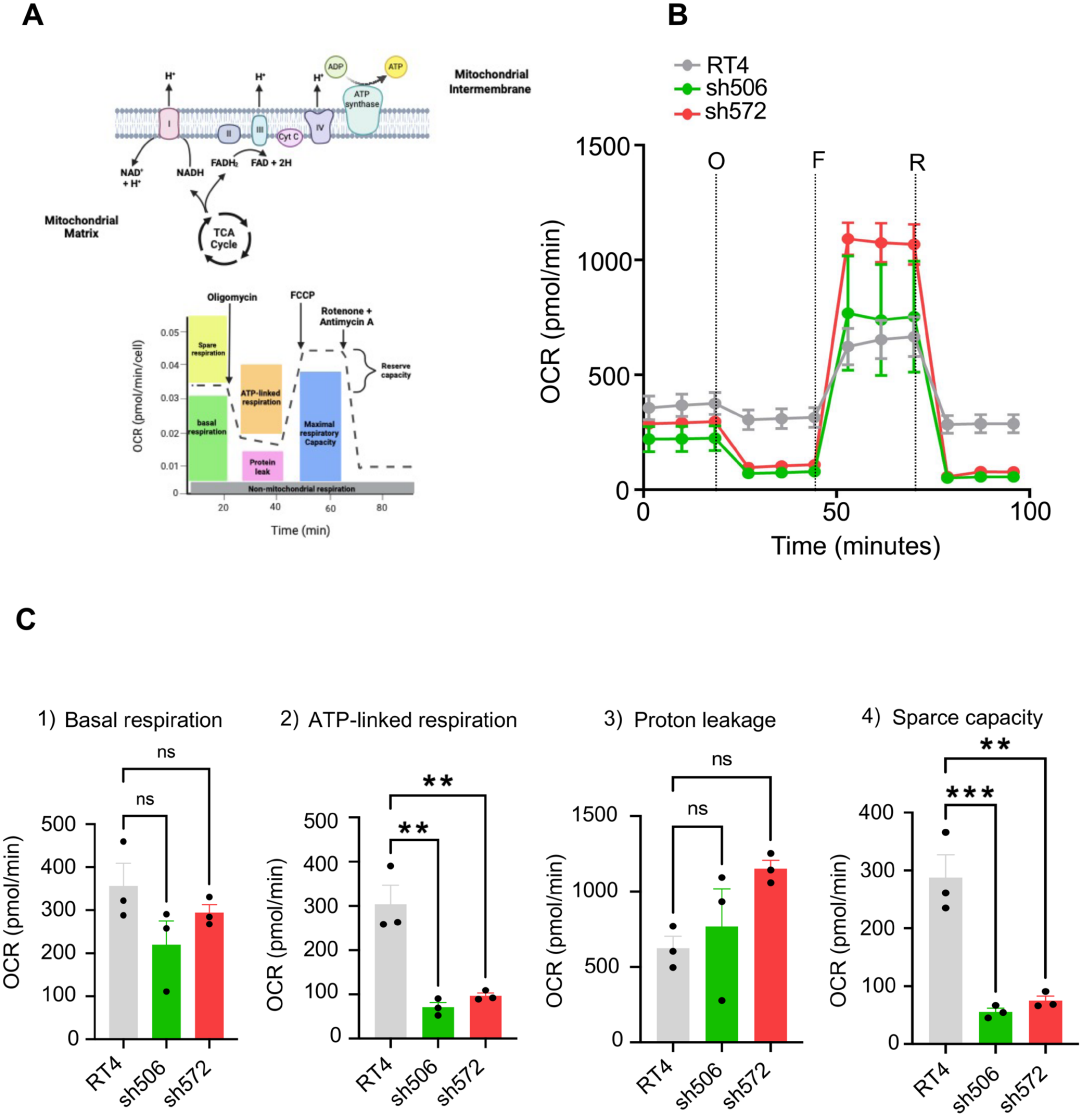
Effect of ALDH1L1 loss on cellular energetics in ALDH1L1‐deficient RT4 clones. (A) Schematic describing the mitochondrial stress test. Schematic was created using BioRender. (B) Differences in OCR between RT4 clones. The OCRs were measured, followed by the sequential treatment with **O** = oligomycin, **F = FCCP**, as well as a mixture of ***R*** = antimycin A and rotenone. Some of the standard error bars are too small and obscured by the symbols representing the mean values. (C) Bar graphs depicting (1) basal respiration; (2) ATP production; (3) maximal respiration; and (4) spare capacity of RT4 clones. Data points represent OCR measurements in triplicate (*n* = 3). Statistical significance is determined by one‐way ANOVA, followed by Tukey's multiple comparison tests (C). ***, *p* < 0.001; **, *p* < 0.01; ns, nonsignificant.

One of the products of the ALDH1L1‐catalyzed reaction is NADPH, indicating that the enzyme likely contributes to cellular energetics. To evaluate cellular respiration in the presence or absence of ALDH1L1, we measured the OCR as depicted in Figure 4A. Data indicate that the basal OCR is similar in the ALDH1L1‐deficient clone (sh572), ALDH1L1‐knockdown clone (sh506), and parental RT4 cells (Figure 4, B and C). ALDH1L1‐deficient (sh572) and ALDH1L1‐knockdown (sh506) clones also had higher responses to oligomycin and a higher maximal respiration than parental cells (Figure 4B,C). Clones sh572 and sh506 showed inhibition of ATP synthase with oligomycin (mitochondrial stress test), and incubation with mitochondrial uncoupler FCCP demonstrated that ALDH1L1‐deficient or knockdown clones display reduced ATP production and reduced maximal mitochondrial respiration, as well as reduced respiratory capacity (the difference between maximal and basal OCR, Figure 4B,C).

### 3.4 Levels of Reduced Folate in RT4 Cells as ALDH1L1 Compensatory Mechanism

ALDH1L1 expression is typically undetectable or very low in most cancer cell lines at both the mRNA (Figure 1A) and protein level [16, 36, 37, 38, 39]. Re‐expression of ALDH1L1 in deficient cells has been shown to exert strong antiproliferative effects [16, 36, 37, 38, 39]. This raises the question of how RT4 cells tolerate high levels of ALDH1L1 without impaired growth. To investigate potential compensatory mechanisms, we evaluated intracellular reduced folate pools in the RT4 cell line. The total folate level in these cells was much higher (150 pmol/mg protein, Figure S3) than we previously reported for A549, HCT116, and HCT‐15 cells (approximately 48, 23, and 17 pmol/mg protein, respectively) [37, 40]. Of note, RT4 cells were cultured in McCoy's 5A medium, which has a 10‐fold higher content of folic acid than the RPMI medium used in our previous studies. As we previously reported, the high folate concentration in the medium results in a significant increase in intracellular folate [41]. High intracellular folate levels also resulted in equally high levels of specific folate coenzymes observed in the present study (Figure S3), which also did not vary between cells with different levels of ALDH1L1 expression. Analysis of the expression of folate pathway enzymes and folate transporters indicates that RT4 cells likely have an enhanced ability to retain folate as polyglutamate derivatives. Thus, levels of two folate transporters, SLC19A1 (RFC1) and SLC46A1 (PCFT), as well as the level of enzyme polyglutamylating folates in the cell, FPGS, were relatively high in RT4 cells (Figure 1C), while levels of two enzymes removing the polyglutamate tail to allow cellular folate export, FOLH1 and GGH, were much lower than average for the analyzed panel (Figure 1C).

### 3.5 ALDH1L1 Depletion Promotes Tumor Growth in RT4 Xenograft Mouse Model

**FIGURE 5:**
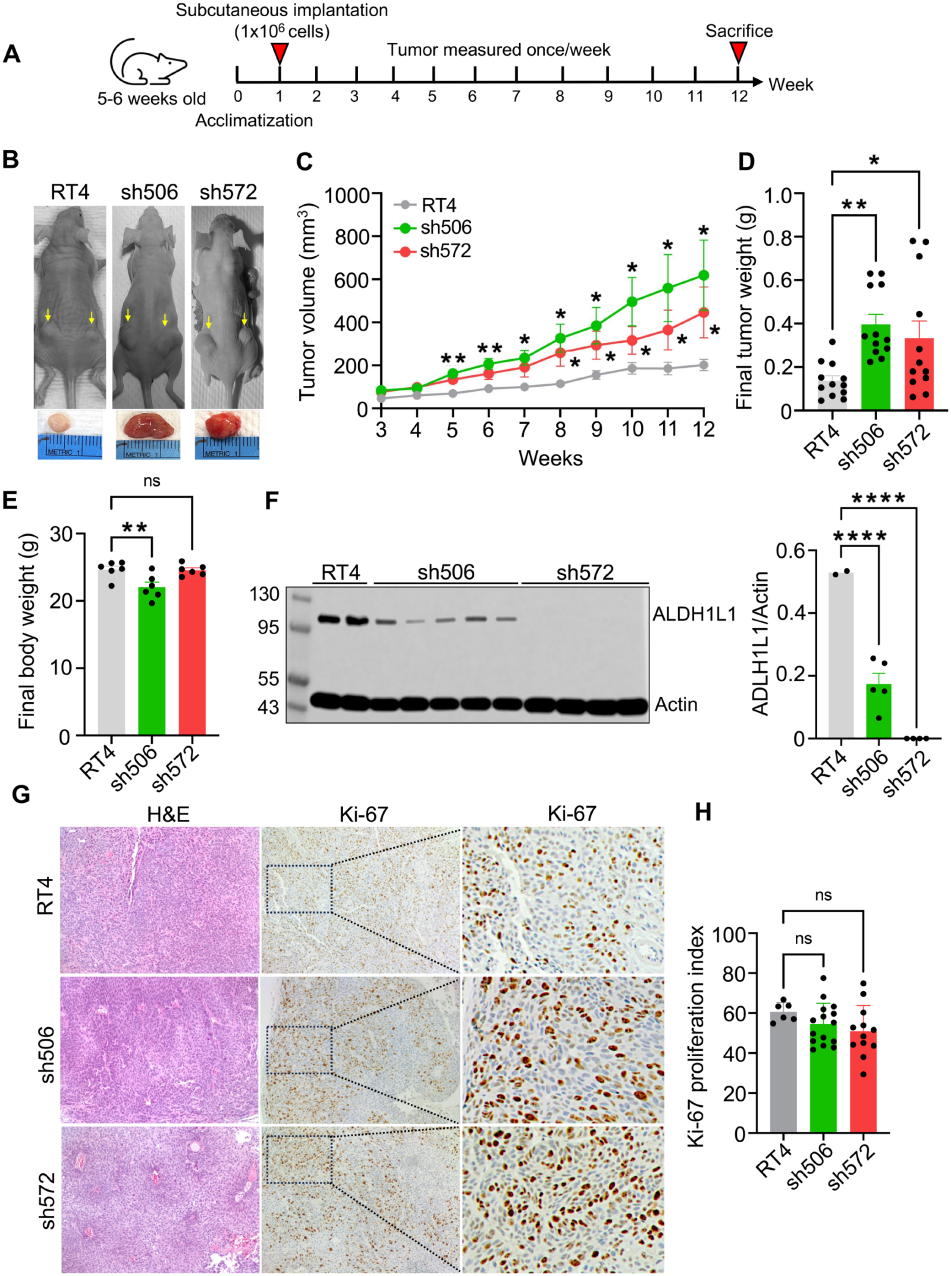
Effect of ALDH1L1 loss on tumor growth in the RT4 xenograft model. (A) Schematic depicting the experimental design. (B) Representative images of mice bearing subcutaneous tumors derived from parental RT4 cells or ALDH1L1‐deficient clones. (C) Tumor volumes recorded throughout the experiment using a digital caliper. Some of the standard error bars are too small and obscured by the symbols representing the mean values. (D) Tumor weights after necropsy (mean ± SEM). (E) Body weights represented as means ± SEM. (F) Left panel, immunoblot assay depicting levels of ALDH1L1 in post‐mortem tumors. Right panel, quantification of ALDH1L1 protein levels in protein bands. (G) H&E (first column) and Ki‐67 IHC staining (middle and last column) of representative parental RT4 and ALDH1L1‐deficient RT4 tumors. (H) Bar graph representing the tumors Ki‐67 IHC staining (mean ± SEM). Statistical significance determined by one‐way ANOVA for comparisons involving more than two groups, followed by Tukey's multiple comparison tests, is denoted as follows: ****, *p* < 0.0001; **, *p* < 0.01; *, *p* < 0.05; ns, nonsignificant (C–F, H).

To assess the role of ALDH1L1 in tumor growth, we established subcutaneous xenograft tumors from RT4 clones in athymic nude mice, as illustrated in Figure 5A. Xenograft tumors were generated in both flanks of nude mice by injecting 2 × 10^6^ cells from each RT4 clone. Tumor growth was monitored for up to 12 weeks (Figure 5A). Our results demonstrated a statistically significant increase in tumor size and weight in ALDH1L1‐deficient groups, sh506 and sh572, compared to the control group derived from parental RT4 cells (Figure 5B–D). Consistent with our findings in cultured RT4 cells, sh506 tumors exhibited significantly greater volume and weight than sh572 tumors (Figure 5B–D). This indicates that even a partial reduction of ALDH1L1 was sufficient to drive a more aggressive tumor phenotype. The final body weights of mice bearing RT4‐derived and sh572‐derived tumors were similar, whereas mice with sh506‐derived tumors showed a slight decrease in body weight compared to controls (Figure 5E). ALDH1L1 expression remained markedly reduced in sh506‐derived tumors, with no detectable expression in sh572‐derived tumors after 12 weeks (Figure 5F). Immunohistochemical staining for Ki‐67 revealed no significant differences in the overall Ki‐67 levels among groups (Figure 5G,H). However, specific tumor regions in sh506‐ and sh572‐derived tumors exhibited more intense Ki‐67 staining than controls (Figure 5G, right panel), suggesting tumor heterogeneity where distinct subpopulations exhibit varying proliferative behaviors.

### 3.6 Metabolomic Profiling of RT4‐Derived Xenograft Tumors

**FIGURE 6:**
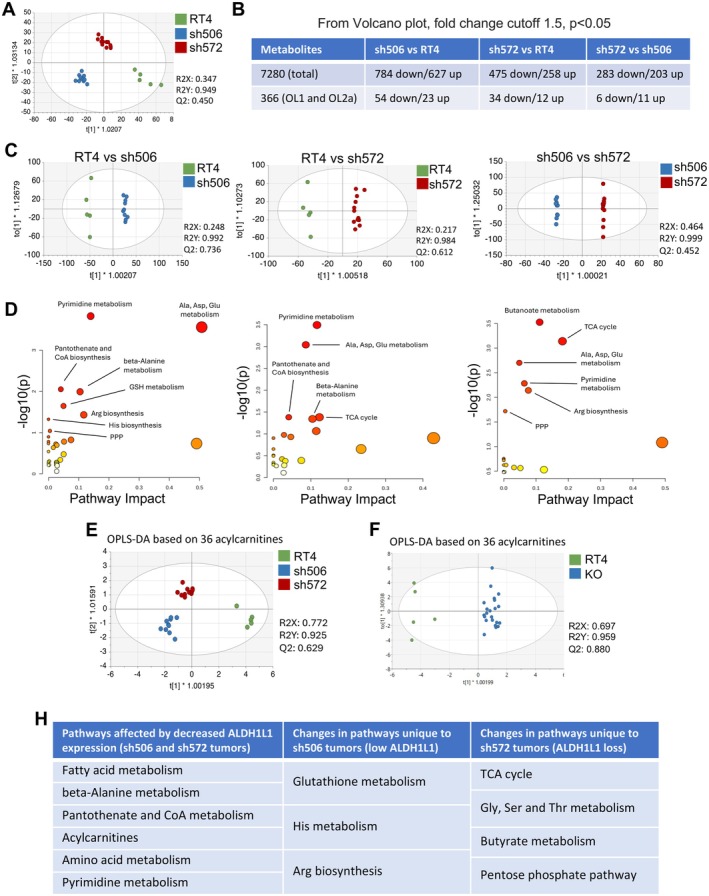
Untargeted metabolomic analysis of RT4‐derived xenograft tumors. (A) OPLSDA of three RT4‐derived tumor groups based on 7280 metabolites. (B) Number of metabolites significantly different between groups (*p* < 0.05; fold change > 1.5). (C) Pairwise OPLSDA between tumors with different ALDH1L1 expression, *left panel*; RT4 versus sh506, middle panel; RT4 versus sh572 and right panel; sh506 versus sh572. (D) Pathway analysis for pairwise tumor group comparisons. (E) OPLSDA of three RT4‐derived tumor groups based on 36 acylcarnitine species. (F) OPLSDA for two group comparison (RT4 and KO/KD) based on 36 acylcarnitine species. Statistics is shown for all OPLSDA plots. (H) Summary of pathways responding to different levels of ALDH1L1 expression (only pathways with the pathway enrichment *p*‐value below 0.05 from panel 6D were included; fatty acid metabolism was included based on the analysis of acylcarnitines).

We have performed untargeted metabolomics analysis of xenograft tumors derived from RT4 cells and sh506 and sh572 clones. This analysis produced 7280 metabolite‐matched features for further investigation (Table S2). Supervised (OPLSDA) analysis of the untargeted metabolomics data showed that the three groups of xenograft tumors (high, low, and undetectable ALDH1L1) with different ALDH1L1 expression levels could be differentiated (Figure 6A). PCA of all study samples did not show clear group separation (S4A,B). Pairwise comparisons of the number of metabolite peaks differentiating groups (using criteria of *p* < 0.05 and absolute fold change > 1.5) indicate that sh506 versus RT4 and sh572 versus RT4 were more different than sh579 versus sh506 (based on the number of altered metabolites, Figure 6B). These data align with our previous report, which demonstrated that in cultured cells, the metabolic differences of sh506 versus RT4 and sh572 versus RT4 were greater than the difference between the two clones [26]. When OPLSDA was applied, strong model statistics (R2Y approaching 1) were demonstrated for the pairwise comparison of the tumor types, showing unique metabotypes for each tumor type (Figure 6C). These results suggest that the extent of ALDH1L1 downregulation has a different effect on tumor metabotype.

Pathway enrichment analysis is shown (Figure 6D) for the comparisons of RT4 versus sh506, RT4 versus sh572, and sh506 versus sh572. Using a cut‐off of −log_10_(*p*‐value) = 1.3 for pathway enrichment significance, the RT4 versus sh506 comparison is characterized by strong differences in pyrimidine metabolism, as well as in alanine, aspartate, and glutamate metabolism, with moderately significant differences in pantothenate biosynthesis, beta‐alanine metabolism, and glutathione metabolism. The RT4 versus sh572 comparison shows similar pathway enrichments for pyrimidine metabolism, weaker enrichments for pantothenate biosynthesis and beta‐alanine metabolism, a smaller impact for alanine, aspartate, and glutamate metabolism, and no impact of glutathione. The TCA cycle pathway enrichment was significant (*p* = 0.041) for the RT4 versus sh572 comparison but not for the RT4 versus sh506 comparison. The comparison of the sh506 versus sh572 pathway enrichment was characterized by strong differences, with a −log_10_(*p*‐value) above 1.3, in butanoate metabolism and the TCA cycle, alanine, aspartate, and glutamine metabolism, pyrimidine metabolism, and arginine biosynthesis.

Examination of the *p*‐values and fold changes of the individual metabolites (Table S2) also showed significant differences in acylcarnitine levels between the phenotypic groups (RT4, sh506, and sh572). OPLSDA (Figure 6E) and PCA (Figure S5A,B), conducted using only peaks that matched 36 acylcarnitines, showed strong model statistics. This analysis indicated that clones with downregulated ALDH1L1 (sh506 and sh572) have more similar acylcarnitine profiles. Therefore, as in our previous study [26], we also analyzed acylcarnitines by combining tumors with decreased and deficient ALDH1L1 expression in a single group (KO) versus the RT4 group. The separation of the two groups (WT vs. KO) was visualized using unsupervised analysis PCA (Figure S5A,B) and OPLSDA (Figure 6F). R2Y and Q2 model statistics were strong (Figure 6F), indicating that acylcarnitines are likely to play a major role in ALDH1L1‐associated metabolic reprogramming. While the total number of peaks in the dataset suggested that there are more differentiating metabolites between WT versus sh506 and WT versus sh572 as compared to sh506 versus sh572 (Figure 6B), we observe that there are still key differences between the two clones, particularly at the pathway level (Figure 6C,D). Overall, our analysis demonstrates similar, as well as unique pathway enrichments when comparing the three different tumor types (summarized in Figure 6H).

## 4 Discussion

Over the past years, reprogramming of cellular metabolism has been underscored as one of the hallmarks of cancer [42]. As part of this reprogramming, one‐carbon metabolism, mediated by folate coenzymes, has attracted significant attention. Cancer cells heavily depend on the availability of one‐carbon groups derived from folate pathways to sustain rapid cell division [15]. These groups are essential for nucleotide synthesis, DNA methylation, and reductive metabolism, processes critical for supporting the high proliferative capacity of cancer cells [43]. Altering the folate cycle in cancer cells through the regulation of one‐carbon metabolizing enzymes can significantly impact tumor growth by enhancing or disrupting the supply of one‐carbon groups toward biosynthetic reactions. In general, the upregulation of several folate‐dependent enzymes promotes cell proliferation, while targeting these enzymes with folate antimetabolites leads to cancer cell death [15, 43, 44, 45].

In this study, we investigated the role of the folate enzyme ALDH1L1 in regulating the proliferation and tumorigenicity of RT4 urinary bladder cancer cells through metabolic reprogramming. While numerous publications and publicly available databases show that ALDH1L1 is ubiquitously silenced in cancer cells, RT4 cells are a rare example of notably high expression of ALDH1L1 [26]. Apparently, these cells have compensatory mechanisms that allow them to tolerate high ALDH1L1 expression. The gene expression profile of RT4 cells aligns with long‐term patient survival, a characteristic feature of low‐grade urothelial carcinomas [24]. From the analysis of mRNA levels for a set of folate‐metabolizing enzymes, we noted that the expression of MTHFD1, which synthesizes the ALDH1L1 substrate 10‐formyl‐THF, and SHMT1 and 2, two enzymes utilizing THF, the product of the ALDH1L1‐catalyzed reaction, is also high in RT4 cells (Figure 1). Thus, high ALDH1L1 in these cells might be needed to prevent the accumulation of 10‐formyl‐THF and supply sufficient THF for the SHMT‐catalyzed reaction of glycine biosynthesis. Two de novo purine pathway enzymes, GART and AICART (ATIC), also utilize 10‐formyl‐THF to yield THF (Figure 1). It is not clear, though, whether the entire purine pathway, which has a total of 11 steps, is sufficiently active in RT4 cells to allow the flux of 10‐formyl‐THF to THF. Notably, RT4 cells have a markedly increased doubling time compared to many other cancer cell lines (36.3 h in our study and 37 h has been reported [46]). As expected, the complete silencing of ALDH1L1 or its strong downregulation in our study significantly enhanced the proliferation of RT4 cells, decreasing the doubling time to about 19.4 and 23.2 h, respectively. As well, colony formation and cell motility were enhanced in ALDH1L1‐deficient clones. These findings align with our previous studies, which demonstrated that the protein knock‐in ALDH1L1‐deficient cancer cells inhibits proliferation and motility [16, 17, 37, 38]. Our previous study in the DEN‐induced model of liver carcinogenesis supported such an effect of the enzyme: ALDH1L1 knockout mice developed larger liver tumors than wild‐type mice at early time points [28]. A recent study from another group demonstrated increased growth of mouse xenograft tumors originating from ALDH1L1‐deficient cancer cells compared to the same cells transfected for ALDH1L1 expression [18].

**FIGURE 7:**
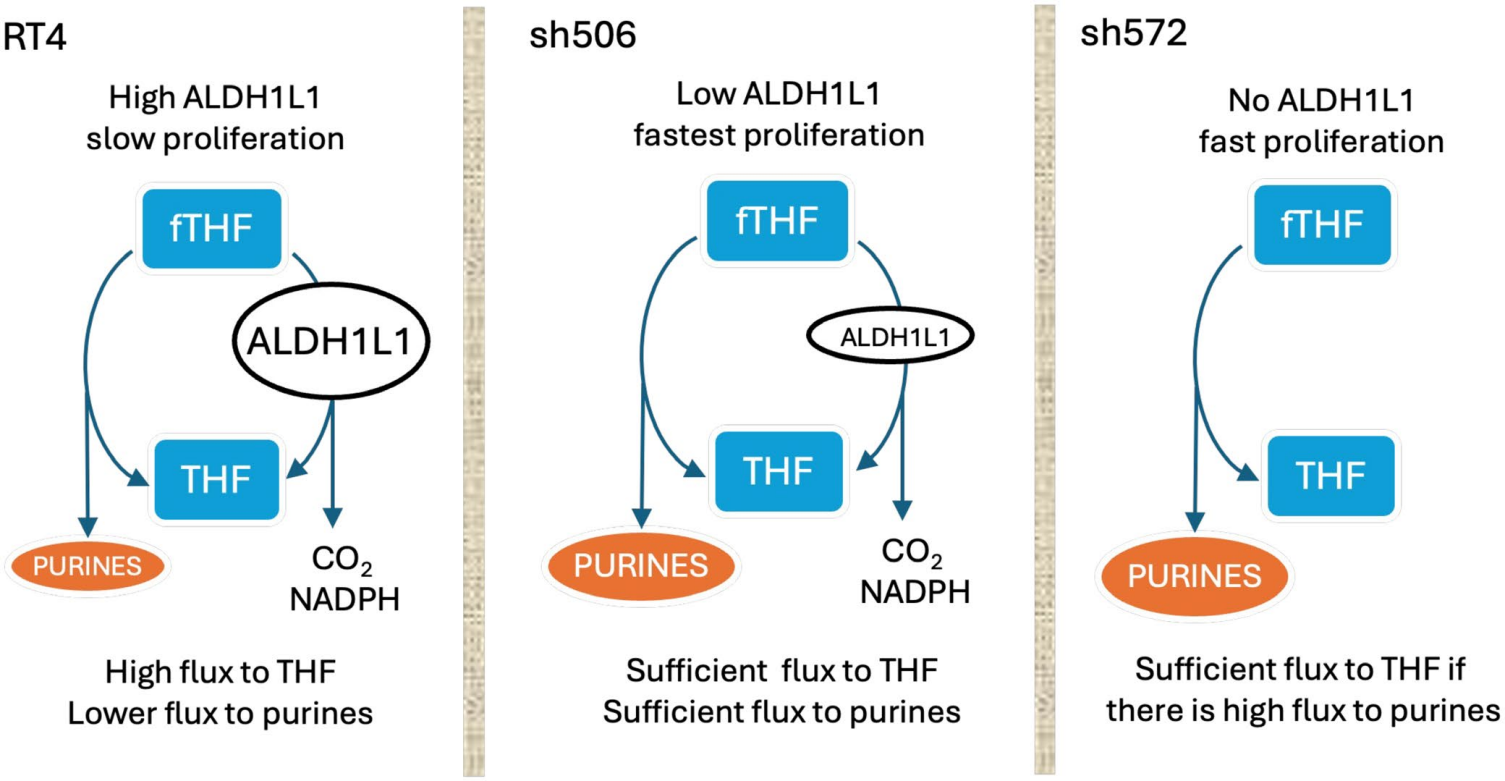
Proposed model for the role of ALDH1L1 in cellular proliferation. ALDH1L1 restores THF pool, which is central to one‐carbon metabolism. This reaction also competes with the de novo purine biosynthesis for the same substrate. In rapidly proliferating cells, ALDH1L1 is downregulated allowing high flux of 10‐formyl‐THF (fTHF) toward de novo purine biosynthesis, which also restores the THF pool. If this purine pathway is moderately active, low expression of ALDH1L1 could be advantageous to restore the THF pool more rapidly.

Here, we employed a reverse approach where ALDH1L1 was silenced in ALDH1L1‐expressing cells. As we predicted, the downregulation of ALDH1L1 in RT4 cells significantly accelerated the growth of xenograft tumors in mice. Interestingly, we have noticed that tumors from the sh506 clone with reduced ALDH1L1 expression grow faster than sh572‐derived tumors with a complete knockout of ALDH1L1. Based on the disposition of the enzyme in folate metabolism, we can offer the following explanation of this phenomenon (schematically depicted in Figure 7). The loss of ALDH1L1 allows the increased flux of one‐carbon groups toward purine nucleotide biosynthesis. Under conditions of low de novo purine biosynthesis, there could be an insufficient generation of THF in ALDH1L1‐deficient cells, which can trap one‐carbon groups such as 10‐formyl‐THF [47]. In this situation, moderate expression of ALDH1L1 could be more beneficial for cancer cells, as it replenishes the THF pool and facilitates other reactions in folate metabolism. Overall, ALDH1L1 is at the intersection of three key pathways: purine biosynthesis, NADPH production, and THF regeneration. Depending on the specific requirements of cells for one pathway versus the other, the decrease of ALDH1L1 expression can be more advantageous for proliferation than its complete loss. In fact, certain cancers still retain the expression of ALDH1L1 [48].

In line with the role of ALDH1L1 in NADPH generation, our study indicates that changes in enzyme expression can impact cellular energetics. This conclusion is based on the evaluation of the OCR, which is widely used to evaluate energy production efficiency [49]. ALDH1L1 loss in RT4 cells does not affect basal OCR but causes a higher response to oligomycin, greater maximal respiration, lower ATP‐linked respiration, reduced spare capacity, and increased proton leak. These findings suggest that ALDH1L1‐deficient cells increase the utilization of energy pathways alternative to mitochondrial ATP production, most likely aerobic glycolysis, which might contribute to enhanced proliferation. Essentially, these cells prioritize division over high levels of mitochondrial ATP production. Such a metabolic shift, known as the Warburg effect, is commonly observed in proliferating cancer cells [50]. Of note, ALDH1L1 has been implicated in maintaining redox balance and mitochondrial function [51, 52]. Overall, ALDH1L1 downregulation promotes metabolic reprogramming that enhances energy production and biosynthesis, fueling the high proliferative capacity of cancer cells.

The link between ALDH1L1 expression and cellular metabolism is intricate, as suggested by our recent untargeted metabolomic analysis of RT4 cells and sh506/sh572 clones [26]. Xenograft tumors metabotype analysis in the present study further supports the notion that the effect of ALDH1L1 on cellular metabolism extends beyond immediate one‐carbon pathways. Four key pathways were altered depending on ALDH1L1 expression levels: (i) alanine, aspartate, and glutamate metabolism; (ii) pyrimidine metabolism; (iii) beta‐alanine metabolism; and (iv) pantothenate and CoA biosynthesis. Additionally, acylcarnitine levels were among the most significantly altered metabolites in tumors underexpressing ALDH1L1 compared to tumors expressing high levels of the enzyme. TCA cycle intermediates were also among the metabolites that strongly depended on ALDH1L1 expression. This metabolic alteration aligns with our previous in vitro study, which demonstrated that RT4 cells exhibited a distinct metabotype depending on the ALDH1L1 expression level [26]. The accelerated tumor growth associated with ALDH1L1 decrease or loss is supported by our study on diethylnitrosamine‐induced hepatocellular carcinoma in mice, where ALDH1L1 knockout enhanced liver tumor progression without affecting tumor initiation or multiplicity [28].

While our study highlights the complex role of ALDH1L1 in tumor progression, it also suggests that downregulation of this enzyme in general is advantageous for malignant growth. Of note, immunohistochemical analyses revealed no difference in the Ki‐67 proliferation index for tumors derived from either the sh506 or sh572 clones versus parental RT4 cells. The lack of change in the Ki‐67 proliferation index following ALDH1L1 knockdown in RT4 cells suggests that tumor expansion in this model may be driven by other mechanisms, possibly through alterations in the tumor microenvironment or through cell behaviors such as migration, size increase, or angiogenesis. Low‐grade papillary urothelial carcinomas, such as those modeled by RT4 cells, exhibit a growth pattern distinct from high‐grade invasive tumors; while displaying “uncontrolled growth” in a structural sense, they do not exhibit unrestrained proliferation, as specific regulatory mechanisms remain intact [53, 54, 55, 56]. It is possible that metabolic reprogramming enables tumor expansion through increased biosynthetic activity and enhanced cellular survival rather than heightened cell division. Alternatively, ALDH1L1‐mediated depletion of one‐carbon units could affect the tumor microenvironment, potentially limiting angiogenesis or stromal support, thereby restricting tumor expansion at a level not reflected by Ki‐67 staining. This phenomenon is consistent with findings in other tumor types, where proliferation rate does not always correlate with tumor aggressiveness or size, suggesting that Ki‐67 alone may not fully capture the biological impact of metabolic alterations in this context [57]. Additionally, tumor heterogeneity arising from genetic, epigenetic, or microenvironment differences can contribute to diverse growth patterns and localized variations in proliferation markers, such as Ki‐67 [58].

## 5 Conclusions

Numerous studies have suggested ALDH1L1's role as a metabolic regulator of cellular proliferation. The apparent basis for such a role is the limitation of one‐carbon groups available for biosynthetic reactions when the enzyme is expressed at higher levels. It is still unclear whether the ALDH1L1 effect is specific to cancer cells. In the present study, we attempted to address the role of ALDH1L1 in tumor progression using RT4 cell‐derived xenograft tumors in nude mice. The selection of this cell line was based solely on a very high ALDH1L1 expression. Thus, our study has limitations. (i) It is unclear which compensatory mechanisms upstream or downstream of the ALDH1L1 pathway allow RT4 cells to tolerate high levels of the enzyme. (ii) RT4 cells, derived from a low‐grade bladder tumor [23, 24, 25], may not recapitulate the characteristics typical of advanced tumors. (iii) The recommended medium for RT4 cells (McCays 5A Modified Media # 16600082) has a high content of folic acid, which did not allow the accurate assessment of the effect of ALDH1L1 on intracellular folates. (iv) Additional strategies for the regulation of ALDH1L1 expression, as well as alternative controls (shRNA) and increased sample sizes, should be considered in follow‐up studies to further validate our findings. Nevertheless, our study concurs with the general idea of ALDH1L1 being a proliferation regulator and a candidate tumor suppressor. Importantly, we compared tumors that completely lack ALDH1L1 with tumors expressing high and low levels of the protein. Untargeted metabolomic analysis of these tumors revealed distinct metabotypes linked to the ALDH1L1 expression and underscored pathways responsive to altered ALDH1L1 levels. Though some metabolic features could be associated with general tumor heterogeneity, it becomes apparent that the ALDH1L1 status has a broad impact on tumor metabolism. Overall, based on metabolomic data from our study, we suggest that ALDH1L1 regulation causes metabolic reprogramming, which may be associated with tumor aggressiveness or responses to chemotherapeutics.

## Author Contributions

**Halle M. Meyers:** conceptualization, methodology, writing – original draft, writing – review and editing, formal analysis. **Jaspreet Sharma:** investigation, methodology, formal analysis, validation, conceptualization. **Amira A. Abdellatef:** conceptualization, investigation, methodology, validation, formal analysis, data curation, writing – original draft, writing – review and editing, visualization. **Mikyoung You:** validation, data curation, formal analysis, methodology. **David Raines:** methodology. **Kyle C. Strickland:** formal analysis, methodology, validation, data curation. **Susan Sumner:** data curation, formal analysis, software, methodology, validation, visualization, writing – review and editing. **Blake R. Rushing:** validation, methodology, software, formal analysis, data curation, visualization, writing – review and editing. **Natalia I. Krupenko:** investigation, writing – review and editing, validation, supervision, conceptualization. **Sergey A. Krupenko:** conceptualization, investigation, validation, data curation, supervision, formal analysis, visualization, writing – review and editing, writing – original draft.

## Conflicts of Interest

The authors declare no conflicts of interest.

## Supporting information


**Figures S1–S5:** cam471291‐sup‐0001‐FiguresS1‐S5.pdf.

**Table S1:** cam471291‐sup‐0002‐TableS1.docx.

**Table S2:** Untargeted metabolomics dataset.

## Data Availability

All data supporting this study are included within the article and/or Supporting Information.
