# Supplementary material for: Differential Expression of One‐Carbon Pathway Enzyme ALDH1L1 Is Linked to Tumorigenicity of Low‐Grade Bladder Cancer Cells Through Metabolic Reprogramming
Source: Cancer Med. 2025 Oct 10;14(19):e71291. doi: 10.1002/cam4.71291 (PMC12512356; doi:10.1002/cam4.71291)
Supplement: Supplementary file 1 — Figures S1–S5: cam471291‐sup‐0001‐FiguresS1‐S5.pdf. [file CAM4-14-e71291-s002.pdf]

Supplementary Figure 1

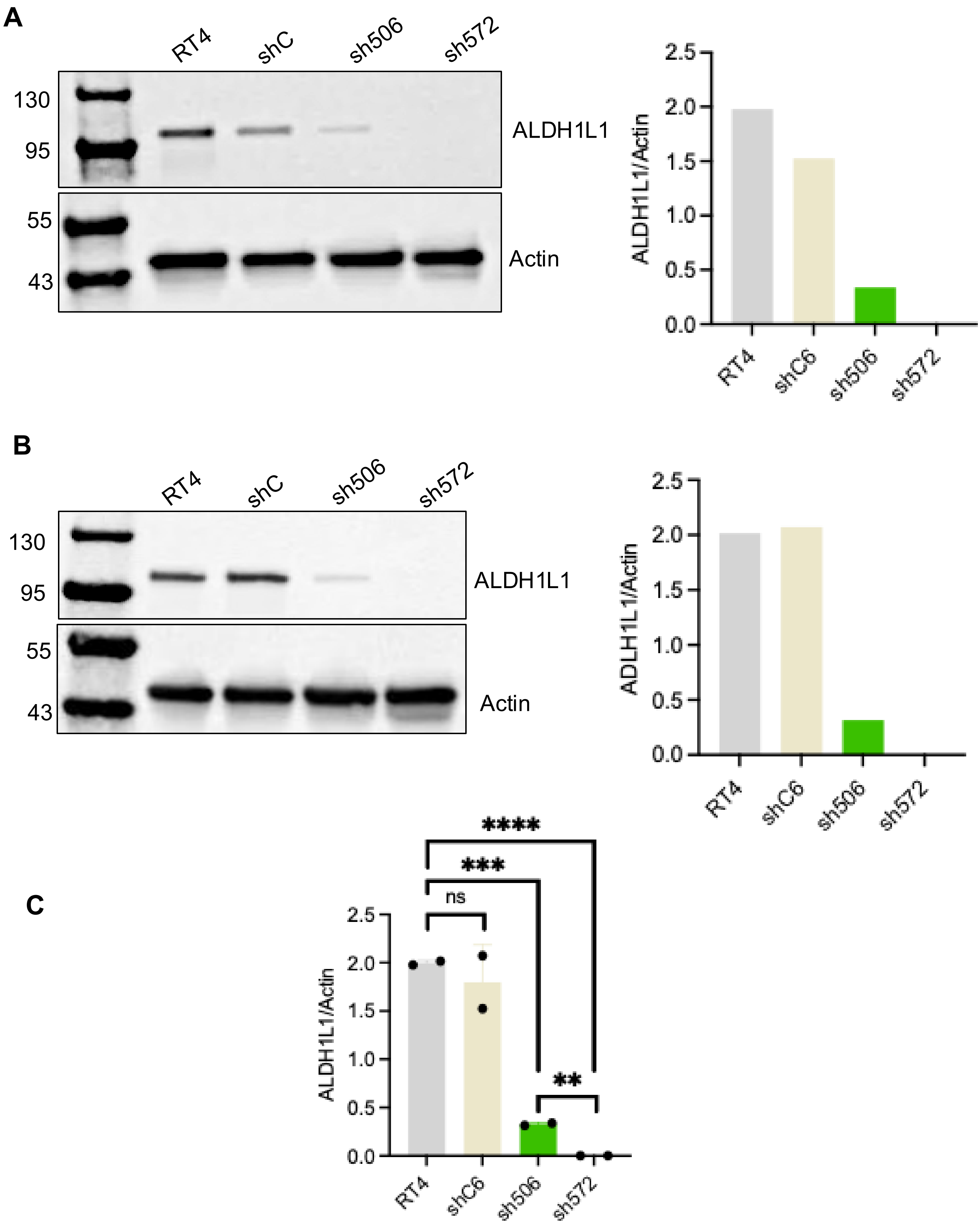

FIGURE S1. Immunoblot analysis showing ALDH1L1 protein levels in RT4 colonies cultured in (A) McCoy's 5A medium (high folate) and (B) RPMI medium (low folate). (C) Quantification of ALDH1L1 band intensities in cells grown under both conditions.

Supplementary Figure 2

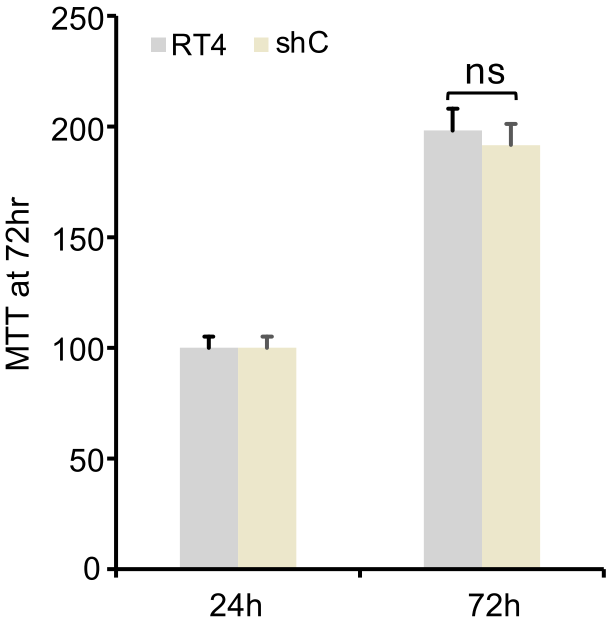

FIGURE S2 MTT assay measuring proliferation at 72 hours, comparing the parental RT4 and shC clone. Data are represented as mean ± SEM of at least three independent experiments. Statistical significance was determined by unpaired Student's t-test after confirming normal distribution.

Supplementary Figure 3

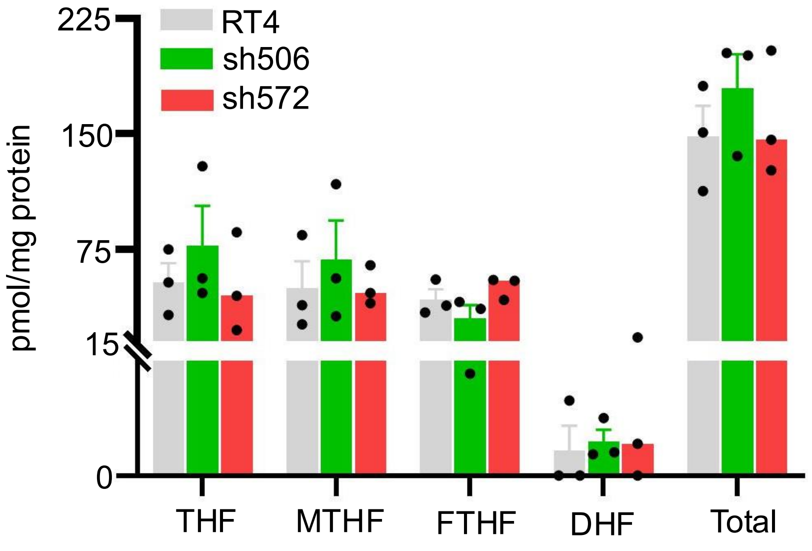

FIGURE S3 Levels of reduced folate pools in parental RT4 cells and ALDH1L1-deficient clones (THF, tetrahydrofolate; MTHF, methyl-tetrahydrofolate; FTHF, formyl-tetrahydrofolate; DHF, dihydrofolate). Data are presented as mean ± SEM (n=3).

Supplementary Figure 4

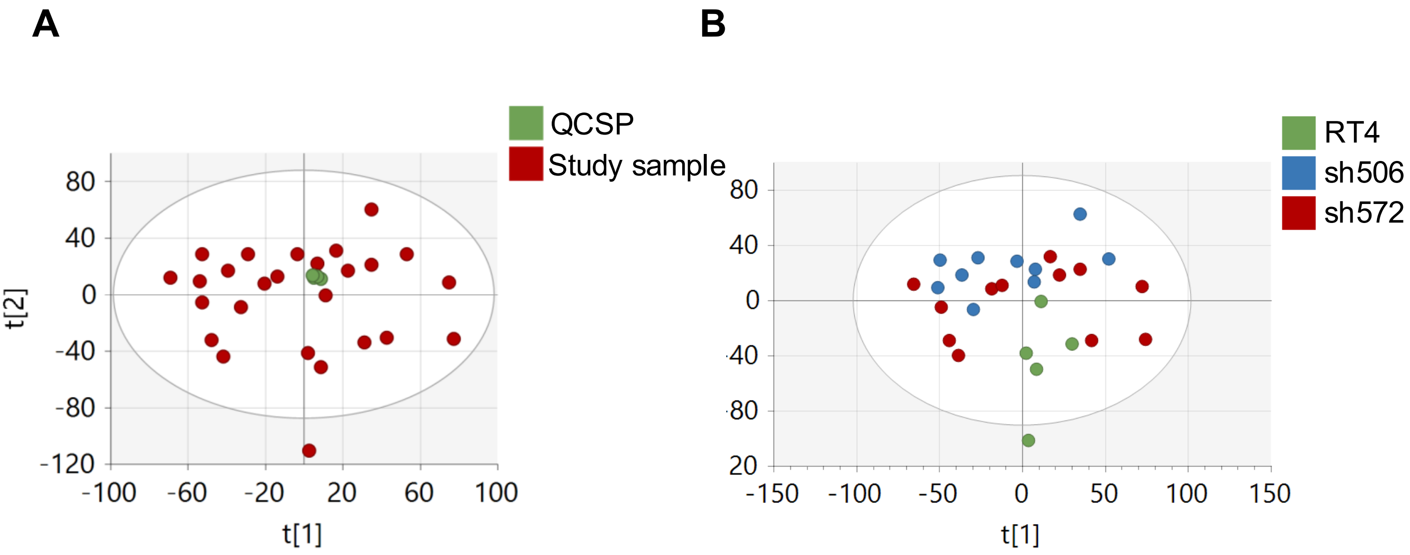

FIGURE S4 (A) Principal component analysis (PCA) of all study samples and quality control study pool (QCSP) samples using all metabolomics features. (B) PCA of study samples only colored by clone.

Supplementary Figure 5

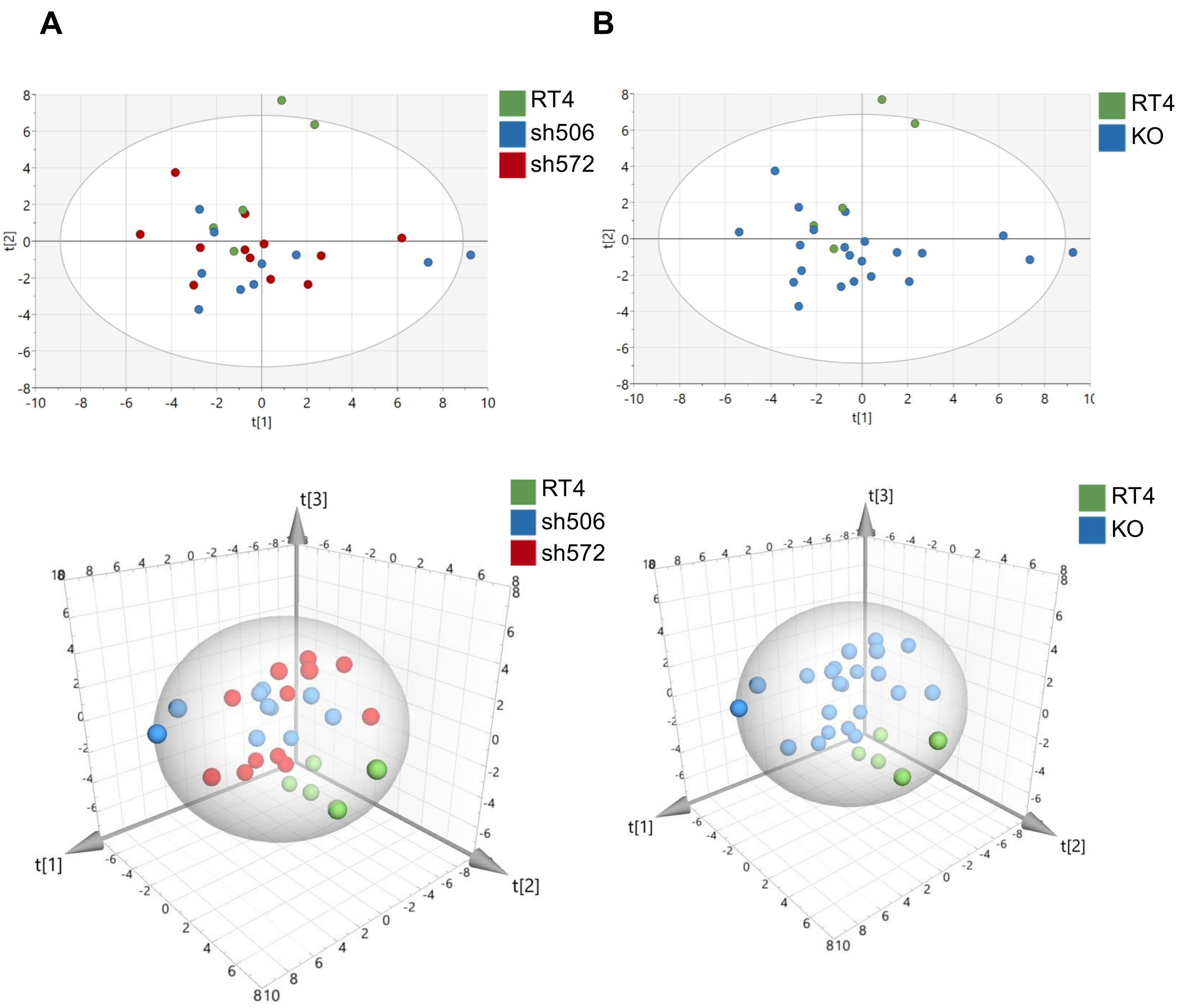

FIGURE S5 PCA plot using 36 acylcarnitines of all study samples colored by clone (A) or by all KO samples combined (B) displayed in two-dimensions (top) and three-dimensions (bottom).
