# Supplementary material for: Differential Expression of One‐Carbon Pathway Enzyme ALDH1L1 Is Linked to Tumorigenicity of Low‐Grade Bladder Cancer Cells Through Metabolic Reprogramming
Source: Cancer Med. 2025 Oct 10;14(19):e71291. doi: 10.1002/cam4.71291 (PMC12512356; doi:10.1002/cam4.71291)
Supplement: Supplementary file 2 — Table S1: cam471291‐sup‐0002‐TableS1.docx. [file CAM4-14-e71291-s001.docx]

**Supplementary table S1** ALDH1L1 mRNA expression in various cancer cell lines, measured as Normalized Transcripts Per Million (nTPM) (<https://www.proteinatlas.org/>)

| Item No. | Cell line | ALDH1L1 mRNA expression (nTPM) | Item No. | Cell line | ALDH1L1 mRNA expression (nTPM) |
| --- | --- | --- | --- | --- | --- |
| **Hepatocellular carcinoma** | | | **Head and Neck cancer** | | |
| 1 | HEP-G2 | 56.1 | 28 | BICR 10 | 31.8 |
| 2 | HuH-1 | 56.1 | 29 | BICR 31 | 31 |
| 3 | SNU-878 | 56 | **Kidney cancer** | | |
| 4 | HuH-6 | 21 | 30 | KMRC-20 | 29.5 |
| **Bladder Cancer** | | | 31 | KMRC-2 | 25.3 |
| 5 | RT4 | **225.3*** | **Leukemia** | | |
| 6 | SW780 | 87.8 | 32 | JVM-3 | 53.5 |
| 7 | UM-UC-1 | 43.5 | **Lung Cancer** | | |
| 8 | HT-1376 | 20.2 | 33 | LOU-NH91 | 30.5 |
| **Esophageal cancer** | | | 34 | NCI-H1836 | 26.7 |
| 9 | JH-EsoAd1 | 78.2 | 35 | NCI-H2110 | 34.1 |
| 10 | TE-10 | 20.2 | 36 | NCI-H596 | 29.9 |
| **Gastric cancer** | | | 37 | PC-14 | 23.3 |
| 11 | KATO III | 93.2 | 38 | RERF-LC-KJ | 60.7 |
| 12 | Okajima | 64.3 | 39 | RERF-LC-Sq1 | 28 |
| 13 | TGBC11TKB | 35.4 | 40 | VMRC-LCD | 25.3 |
| 14 | IM95 | 30.4 | **Sarcoma** | | |
| **Colorectal Cancer** | | | 41 | HT-1080 | 21.3 |
| 15 | SNU-1040 | 75.2 | **Pancreatic cancer** | |  |
| 16 | SW48 | 58.6 | 42 | PK-8 | 175.2 |
| 17 | LS180 | 39.4 | **Ovarian Cancer** | | |
| 18 | SW837 | 26.5 | 43 | OVCAR-5 | 40 |
| 19 | SNU-407 | 32.5 | 44 | HCC630 | 27.2 |
| 20 | SNU-C2A | 25 | **Myeloma** | | |
| 21 | GP2d | 38.5 | 45 | Karpas-707 | 77.9 |
| **Breast Cancer** | | | 46 | KE-97 | 65.6 |
| 22 | AU565 | 24.6 | 47 | HuNS1 | 39.3 |
| 23 | BT-20 | 52.6 | 48 | U-266/70 | 42.2 |
| 24 | JIMT-1 | 25 | **Lymphoma** | | |
| **Brain Cancer** | | | 49 | EHEB | 41 |
| 25 | LN-464 | 23.4 | 50 | BCP-1 | 32 |
| **Bone Cancer** | | | 51 | L-540 | 32.8 |
| 26 | A-673 | 31.6 | 52 | OCI-Ly10 | 44 |
| 27 | RD-ES | 37.5 | 53 | SCC-3 | 23.3 |

*****RT4 cells exhibited the highest expression of ALDH1L1 when compared to all other tested cell lines.
